# Nicotinamide benefited amino acid metabolism and rumen fermentation pattern to improve growth performance of growing lambs

**DOI:** 10.5713/ab.24.0015

**Published:** 2024-08-22

**Authors:** YuAng Wang, Hao Wu, Yiwei Zhang, Mingfeng Fei, Zhefeng Li, Daxi Ren, Chong Wang, Xiaoshi Wei

**Affiliations:** 1 College of Animal Science and Technology, College of Veterinary Medicine, Zhejiang A & F University, Zhejiang, 311300, China; 2 Huzhou Zifeng Ecological Agriculture Co., Ltd, Huzhou, Zhejiang 313000, China; 3 Hangzhou King Techina Feed Co., Ltd, Hangzhou, Zhejiang 311107, China; 4 Institute of Dairy Science, College of Animal Science, Zhejiang University, Hangzhou, Zhejiang, 310058, China

**Keywords:** Amino Acid Metabolism, Antioxidant Capacity, Metabolomics Profile, Rumen Fermentation Pattern, Rumen-protected Nicotinamide

## Abstract

**Objective:**

Nicotinamide (NAM) is easily degraded in the rumen, but the rumen-protected NAM (RPN) supplementation might enable the use of NAM in ruminants. This study aimed to elucidate the effects of RPN supplementation on growth performance, rumen fermentation, antioxidant status and amino acid (AA) metabolism in growing lambs.

**Methods:**

A total of 128 healthy and similar lambs (21.3±0.28 kg, 70±6.3 days of age) were allotted to 1 of 4 groups. The treatments were 0, 0.5, 1, and 2 g/d RPN supplementation. The RPN products (50% bioavailability) were fed at 0700 h every day for 12 weeks. All lambs were fed the same pelleted total mixed rations to allow *ad libitum* consumption and had free access to water.

**Results:**

The RPN tended to increase the average daily gain and feed efficiency. The tendencies of RPN×day interaction were found for dry matter intake during the entire study (p = 0.078 and 0.073, respectively). The proportions of acetic acid, isobutyric acid and isovaleric acid were decreased, whereas the proportions of propionic acid and valeric acid were increased (p<0.05). The ratio of acetic acid to propionic acid was decreased (p<0.05). Moreover, the antioxidative status was enhanced and the glucose concentration was increased by RPN (p<0.05). In addition, 17 AAs were detected in plasma, of which 11 AAs were increased by RPN (p<0.05). Plasma metabolomics analysis identified 1,395 compounds belonging to 15 classes, among which 7 peptides were significantly changed after RPN supplementation.

**Conclusion:**

Overall, the results suggested that RPN supplementation favoured the rumen fermentation pattern to propionic acid-type with benefited glucose metabolism, enhanced antioxidant capacity, and changed the AA and small peptide metabolism. This study provides a new perspective for studying the relationship between vitamin and AA metabolism.

## INTRODUCTION

Nicotinamide (NAM) is a precursor for the synthesis of nicotinamide adenine dinucleotide (NAD) and nicotinamide adenine dinucleotide phosphate (NADP) and widely participates in redox reactions [1]. NAM supplementation was previously suggested to benefit energy metabolism by regulating glucose and lipid metabolism, thus increasing ATP production in dairy cows and goats [2–5]. Maternal NAM supplementation also inhibited lipolysis in offspring kids [6,7]. Whether accelerated energy metabolism and reduced lipolysis could benefit growth performance is unclear. In addition, the serum metabolomics profile indicated that arginine and proline metabolism was enriched in dairy cows supplemented with NAM [3], while specific changes in amino acids (AAs) were not detected.

The rumen is an important digestive organ in ruminants and can provides most of the required energy by the host. NAM and nicotinic acid (NA) are different forms of vitamin B_3_, and supplemented NAM and NA would be degraded in the rumen, leaving only a small portion to flow into the small intestine [8]. Thus, supplementation with high doses of NAM [2] and NA [9,10], and rumen-protected forms of NA [11–13] were proven to be bioeffective. However, the rumen-protected form of NAM has not been applied before.

Based on these results, we hypothesized that i) NAM supplementation could improve the production performance of growing lambs, and ii) NAM could elicit changes in lipid and AA metabolism. To provide more bioactive and accurate NAM, rumen-protected NAM (RPN) was used in this study. Hu sheep have become popular and important in China due to its excellent prolificacy (3 to 4 lambs per parturition), rapid growth rate, and adaptation to poor-quality feeds. Thus, Hu lambs were used as experimental subjects. The objectives of this study were not only to determine the effects of RPN on the production performance of growing lambs but also to profile the changes in blood metabolites.

## MATERIALS AND METHODS

This study was conducted in accordance with the guidelines of the Institutional Animal Care and Use Committee of Zhejiang A&F University of China (Hangzhou) (ZAFUAC 2021089). Hu lambs at a commercial farm in Zhejiang Province of China were used.

### Animals, feed, and experimental design

One hundred and twenty-eight healthy male Hu lambs with similar body weight (BW) (21.3±0.28 kg) and age (70±5.3 days) were randomly allotted to 1 of 4 treatments, with each group having 32 growing lambs. The 4 treatment groups were named based on the supplemental dose of RPN, including T_0_ (no RPN supplementation), T_0.5_ (supplemented with 0.5 g/d RPN), T_1_ (supplemented with 1 g/d RPN), and T_2_ (supplemented with 2 g/d RPN). The RPN products (containing 60% NAM, with a rumen bypass ratio of 83.6%; Hangzhou King Techina Feed Co., Ltd, Hangzhou, China) were fed at 0700 h every day. The doses of RPN used in the present experiment were determined based on the metabolic BW conversion between Hu sheep and dairy goats, as shown in our previous study [6]. All lambs were fed the same pelleted total mixed rations to allow *ad libitum* consumption and had free access to water. The commercial pelleted total mixed rations consisted of corn, soybean meal, distillers dried grains with soluble, groundnut stem meal, limestone, salt, vitamins, minerals, rumen-protected AAs, probiotics, phytase and preservatives. The nutrition analyses are shown in Table 1. The animal experiment lasted for 12 weeks.

### Dry matter intake and body weight of lambs

The amounts of pellet offered and refused were recorded weekly using an automatic feeding recorder. The samples of pellet were collected and dried at 65°C for 48 h, and dry matter (DM), crude protein (CP), ether extract, neutral detergent fibre, acid detergent fibre, and starch were measured as described previously [2]. The BW of individual lamb was recorded every month (days 70, 100, 130, and 160 of age) before the morning feeding. The average daily gain (ADG) was calculated based on the BW, and the feed efficiency was calculated as the ratio of ADG to DMI.

### Collection and analyses of rumen fluid

At end of experiment, ten lambs were randomly selected from every group, and rumen fluid was collected at 1500 h. The ruminal fluid from lambs was collected using an oral rumen fluid sampler and the first 150 to 200 mL of ruminal content was discarded to avoid saliva contamination. Approximately 100 mL of ruminal fluid was collected and strained through a 2-layer cheesecloth [14]. One millilitre of 25 g/100 mL metaphosphoric acid was added to 4 mL of filtrate to avoid dissociation of acid, and the volatile fatty acids (VFAs) was analysed using gas chromatography according to a previous methodology [15]. The isoacids were calculated as the sum of isobutyric acid, isovaleric acid and valeric acid. Ammonia nitrogen (NH_3_-N) was determined according to Hu et al [16], and microbial protein (MCP) was measured according to the purine method [17].

### Collection and analyses of blood

Ten blood samples were collected from the lambs selected at the end of experiment. Approximately 10 mL blood samples were collected 3 h after morning feeding from the jugular vein into individual heparinized tubes. Blood was centrifuged at 3,500 g and 4°C for 15 min to obtain plasma and then stored at −80°C.

The plasma total antioxidant capacity (T-AOC), activities of superoxide dismutase (SOD) and catalase (CAT), malondialdehyde (MDA) concentration, glucose, triglycerides (TG), total cholesterol (TC), nonesterified fatty acids (NEFA), and blood urea nitrogen (BUN) were analysed using enzymatic methods in an autoanalyzer according to our previous reports [2,7]. The blood ammonia concentration and diamine oxidase (DAO) activity were determined using commercial kits (Jiancheng Bioengineering Institute, Nanjing, China). Plasma D-lactate was measured using enzyme-linked immunosorbent assay kits (Bangyi, Shanghai, China). The plasma NAM concentration was measured using the ELISA method (sheep catalog no. TW52104H; Shanghai Tongwei Industrial Co., Ltd., Shanghai, China). The concentrations of plasma AAs were determined using reversed-phase high-performance liquid chromatographic (HPLC) after derivatization with o-phthaldialdehyde [18].

### Metabolome composition

Based on the results of production performance, rumen fermentation and plasma parameters, the plasma samples from the T_0_ and T_1_ groups were used to profile the changes in metabolites and explore the potential mechanism for the changes. The metabolites of the plasma sample (50 μL) were extracted using 300 μL of 20% acetonitrile methanol internal standard extractant. The mixture was vortexed for 3 min and centrifuged (13,400 g, 4°C) for 10 min. Then, 200 μL of the supernatant was transferred and incubated at −20°C for 30 min. The mixture was centrifuged (13,400 g, 4°C) for 3 min, and 150 μL of supernatant was removed for analysis using a high-performance liquid chromatography-electrospray tandem mass spectrometry system (UPLC, ExionLC AD; MS, QTRAP 6500 + System; Sciex, Framingham, MA, USA). The quality-control sample was prepared by mixing equal volumes of all samples. The analytical conditions were as follows: UPLC: column, Waters ACQUITY UPLC HSS T3 C18 (1.8 μm, 2.1×100 mm); column temperature: 40°C; flow rate: 0.4 mL/min; injection volume: 2 μL; solvent system: water, acetonitrile; gradient program, 95:5 V/V at 0 min, 10:90 V/V at 10.0 min, 10:90 V/V at 11.0 min, 95:5 V/V at 11.1 min, and 95:5 V/V at 14.0 min. QTRAP LC-MS/MS System, equipped with an electrospray ionization (ESI) Turbo Ion-Spray interface, operating in positive and negative ion mode and controlled by Analyst 1.6.3 software (Sciex, USA). The ESI source operation parameters were as follows: source temperature 500°C; ion spray voltage (IS) 5,500 V (positive), 4,500 V (negative); ion source gas I (GSI), gas II (GSII), curtain gas (CUR) was set at 50, 50, and 25.0 psi, respectively; the collision gas (CAD) was high. Data collection and analysis were conducted with the free online platform from Metware Cloud Platform ( https://cloud.metware.cn/#/home ).

### Statistical analyses

The MIXED procedure of SAS was used for data analyses. The methods followed the procedures we previously reported in Wei et al [7]. The REPEATED procedure with first order autoregressive covariance structure was used for DMI and BW of lambs, as they were measured repeatedly. Effects on BW, ADG, and feed efficiency for specific days, and rumen fermentation and blood parameters were analyzed using the linear model, and the adjusted Tukey test was implemented to compare the differences between means. In addition, polynomial contrasts were used to compare the linear and quadratic effects of 3 incremental levels of RPN versus the control. The difference was considered significant at p≤0.05 and as a trend at 0.05<p≤0.10.

## RESULTS

### BW, DMI, and production performance

**Figure 1:**
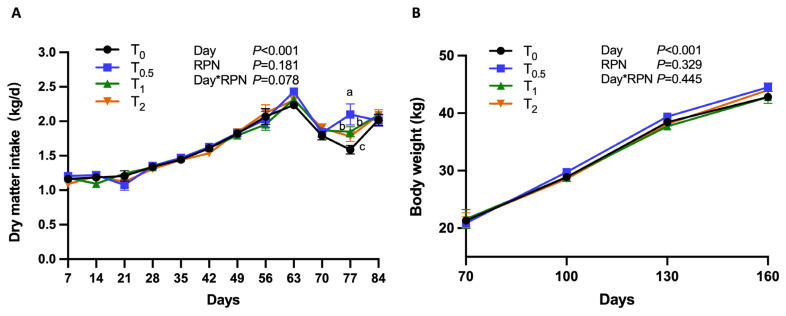
The dry matter intake (A) and body weight (B) of the growing lambs. T_0_, T_0.5_, T_1_, and T_2_ represent growing lambs supplemented with 0, 0,5, 1, and 2 g/d rumen-protected nicotinamide, respectively. ^a–c^ Different letters denote significant difference p<0.05.

The growth performance of growing lambs is shown in Table 2 and the trends of DMI and BW over time is shown in Figure 1. The DMI was increased in all T_0.5_, T_1_, and T_2_ groups at d 77, and the DMI in T_0.5_ was higher (p<0.05; Figure 1A). We also detected a tendency for a day×NAM interaction to affect DMI (p = 0.078). The BW was not affected by RPN at any measurement or during the entire study or by the interaction effect of day×NAM. Tendencies were found for ADG at days 70 to 130 and feed efficiency at days 130 to 160 (p = 0.065 and 0.066, respectively).

### Ruminal fermentation characteristics

The effect of different doses of RPN on rumen fermentation is presented in Table 3. The total VFA concentrations were increased in all T_0.5_, T_1_, and T_2_ groups (p<0.05), and that was much higher in T_2_ group (p<0.05). As expressed as a percentage of total VFAs, ruminal acetic and isobutyric acid were decreased in all T_0.5_, T_1_, and T_2_ groups (p<0.05). Propionic acid was increased, while isovaleric acid was decreased in both the T_1_ and T_2_ groups (p<0.05). Ruminal valeric acid was increased in all T_0.5_, T_1_, and T_2_ groups (p<0.05). The ratio of ruminal acetic acid to propionic acid was decreased in the T_1_ group (p<0.05) compared with the T_0_ group. The proportions of isoacids, ruminal NH_3_-N and MCP were similar among groups.

### Plasma indicators and amino acid concentrations

The plasma parameters related to antioxidants, lipid metabolism and intestinal health are shown in Table 4. The NAM concentration was increased in all T_0.5_, T_1_, and T_2_ groups (p<0.05), and that was much higher in T_2_ group (p<0.05). The T-AOC was found to be increased in both the T_0.5_ and T_1_ groups (p<0.05), and the activity of SOD was elevated in both the T_1_ and T_2_ groups (p<0.05). The CAT and MDA were not affected by RPN. The glucose concentration was increased in both the T_1_ and T_2_ groups (p<0.05), and the indicators related to lipid metabolism (TG, TC, and NEFA) were not changed. Total protein was increased by RPN only in the T_1_ group (p<0.05), and BUN was similar among the groups. In addition, blood ammonia was higher in the T_1_ group (p<0.05), and D-lactate and DAO were similar.

In total, 17 AAs were detected in the plasma of lambs, and 11 AAs were found to be increased by RPN in this study (p< 0.05, Table 5). The concentrations of asparagine, threonine, glutamine, and arginine were increased in the T_2_ group (p< 0.05). The concentrations of serine, proline, glycine, isoleucine, phenylanaline, lysine, and total AAs were increased in both the T_1_ and T_2_ groups (p<0.05), of which the serine in the T_2_ group was higher than that in the T_1_ group (p<0.05). The valine concentration was increased only in the T_1_ group (p<0.05).

### Plasma metabolome profile

**Figure 2:**
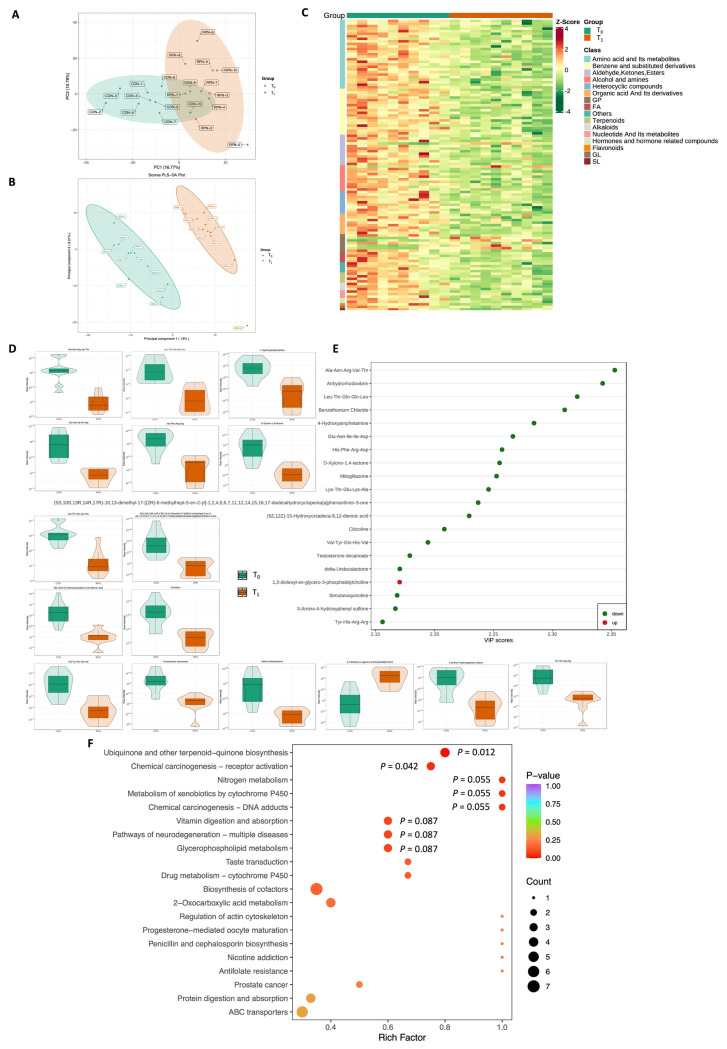
The metabolome profile in the plasma of growing lambs supplemented with rumen-protected nicotinamide. The significantly different metabolites based on the variable importance for the projection (VIP) >2 and p<0.05. (A) Principal component analysis (PCA)-2D, (B) Partial least squares-discriminant analysis (PLS-DA), (C) Hierarchical clustering analysis, (D) Violin plot of each significantly different metabolite peak area (Log10 transformed) of plasma metabolites between T_0_ and T_1_, (E) The top 20 significantly different metabolites with names, and (F) The enriched Kyoto encyclopedia of genes and genomes pathways of the more significantly different metabolites between T_0_ and T_1_. T_0_ and T_1_ represent growing lambs supplemented with 0 and 1 g/d rumen-protected nicotinamide, respectively.

After quality control, 1,395 metabolites in plasma were identified and quantified. The trends of distinction between T_0_ and T_1_ were observed according to the principal component analysis based two-dimensional (PCA-2D) (Figure 2A) and partial least squares-discriminant analysis (PLS-DA) maps (Figure 2B). The heatmap shows clear differences between T0 and T1 based on the top 100 metabolites (Figure 2C). In addition, 16 significantly different metabolites were identified between T_0_ and T_1_ based on the variable importance for the projection (VIP) >2 and p<0.05 (Figure 2D; 2E). Seven small peptides, including Ala-Asn-Arg-Val-Thr (VIP = 2.35; p<0.001; fold change = 1.29), Leu-Thr-Gln-Gln-Leu (VIP = 2.32; p<0.001; fold change = 1.63), Glu-Asn-Ile-Ile-Asp (VIP = 2.27; p<0.001; fold change = 1.25), His-Phe-Arg-Asp (VIP = 2.26; p<0.001; fold change = 1.29), Lys-Thr-Glu-Lys-Ala (VIP = 2.25; p<0.001; fold change = 1.55), Val-Tyr-Gln-His-Val (VIP = 2.19; p<0.001; fold change = 1.69), and Tyr-His-Arg-Arg (VIP = 2.16; p<0.001; fold change = 1.67), were higher in T_1_ than in T_0_ (Figure 2D). The enriched metabolism pathways were involved in ubiquinone and other terpenoid-quinone biosynthesis (p = 0.012), chemical carcinogenesis-receptor activation (p = 0.042), nitrogen metabolism (p = 0.055), metabolism of xenobiotics by cytochrome P450 (p = 0.055), chemical carcinogenesis-DNA adducts (p = 0.055), vitamin digestion and absorption (p = 0.087), pathways of neurodegeneration-multiple diseases (p = 0.087), and glycerophospholipid metabolism (p = 0.087) (Figure 2F).

## DISCUSSION

This experiment was designed to test the hypothesis that RPN supplementation could accelerate the efficiency of nutrient oxidation and metabolism, thus improving the production performance of growing lambs. The results varied and partially supported this hypothesis.

In growing lambs, the RPN did not change the BW in this study. There are some possible reasons for the unchanged BW, such as the healthy status of animals, the requirement for NAM, and experimental conditions of the study, etc. The effect of NAM on BW of animals is worth exploring further. We found the RPN tended to increase DMI and decrease feed efficiency during the experimental period. These results might be due to the changes in rumen fermentation and metabolism efficiency, as we discussed below, further supporting the supplementation of RPN during the growing period.

Intelligent microcapsule technology is widely applied in nutrient supplementation that needs to be protected in the rumen then released mainly in the intestine. The rumen bypass ratio is always lower than 100%. The NAM was rumen protected in this study, and the rumen bypass ratio of RPN was 83.6%, indicating that there would be some NAM released and digested in the rumen. Previous studies have shown that the rumen fermentation and metabolism would be altered although using rumen protected products [19,20]. In total, rumen fermentation was promoted by RPN, as the concentrations of total VFAs and almost all kinds of VFAs we detected were increased in this study. The RPN supplementation would induce partial digestion of NAM and protected substances in the rumen. This result was consistent with our previous study in dairy goats that were supplemented with NAM in intact form [4]. In addition, the ruminal microbiota profile (data unpublished) could contribute to the rumen fermentation.

For ruminants, acetic acid is the main precursor for the de novo synthesis of fatty acids and provides most of the carbon. The concentration of ruminal acetic acid was increased by RPN. Ruminal infusion of acetate could increase milk fat yield in lactating dairy cows [21]. In this study, whether the fatty acids in muscle and adipose tissue were altered needs further validation. Even when the concentration of acetic acid was increased, the proportion of acetic acid was decreased by RPN. The ruminal proportion of propionic acid was increased with RPN supplementation. Similar results were found by Samanta et al [22] and Khan et al [23], in which the proportion of propionic acid and concentration of total VFAs were increased with NA supplementation, and the increases might be related to the NADH/NAD ratio. Propionic acid is the main source of liver gluconeogenic substrate, suggesting that RPN might improve gluconeogenesis by increasing ruminal propionic acid, and the elevated plasma glucose concentration in this study might support this. In addition, the decreased ratio of acetic acid to propionic acid indicated that the rumen fermentation pattern was altered to propionic acid-type, contributing to the production of more substrates for energy metabolism. As reported in our previous study [4], using a high dose of the unprotected form of NAM, we also found that NAM increased the proportion of propionic acid and valeric acid and decreased the ratio of acetic acid to propionic acid suggesting that the alteration in rumen fermentation was caused by NAM supplementation.

The proportion of isoacids was not altered, while the proportions of isobutyric acid and isovaleric acid were decreased, and the proportion of valeric acid was increased in this study. Isoacids are the sum of isobutyric acid, isovaleric acid and valeric acid, which are mainly derived from oxidative deamination and decarboxylation of feed branch chain AA by microorganisms. The NAM, as a vitamin B, was absorbed to be digested by rumen microbes for growth and was also produced by them, indicating that supplementation with RPN could elicit effective fermentation of feed in the rumen, possibly due to microbial growth. The microbial fermentation of cellulose and hemicellulose is the main source of VFAs in the rumen. An increased microbial population may lead to increased MCP flows and intestinal AA supply, while the MCP was similar among groups. Thus, further research is warranted to investigate the effect of RPN on rumen microbiota.

The RPN supplementation elevated the antioxidant capacity and blood glucose concentration in growing lambs, which was in accordance with our previous findings in dairy cows and goats [2–4]. Thus, we shall not discuss it here. Unlike findings in our previous studies, the RPN did not change the plasma lipid parameters, such as TG, TC, and NEFA, in this study. One possible reason for the differences would be the different physiological stages. During the peripartum period, the blood concentrations of NEFA and TG were higher due to lipolysis occurring as a response to the negative energy balance. The NAM was proven to enhance lipogenesis and decrease lipolysis of adipose tissue [24,25]; thus, blood lipid indicators were decreased [2]. However, for fattening lambs, the lipolysis process would be lower. Enhancing lipogenesis might not cause the changes in blood parameters. The unchanged D-lactate and DAO indicated that RPN had no effect on the intestinal barrier or health.

The most interesting finding of our study would be the alteration in AA metabolism by RPN supplementation. The effect of feeding RPN or NAM on plasma AA composition in ruminants has not been reported before. In a study by Jones et al [26], NAM pretreatment resulted in a significant increase in AA uptake and catabolism into malate in leukemia stem cells, inhibition of NAM phosphoribosyltransferase, the rate-limiting enzyme in NAM metabolism, and decreased AA and fatty acid metabolism. This was based on the role of NAD as an upstream driver of both AA and fatty acid metabolism, which also resulted from decreased TCA cycle enzyme activity [27]. Usually, when supplementing AAs, such as lysine [28], methionine [29], and leucine [30], blood AA concentrations are increased in ruminants. Previously, we found that arginine and proline metabolism was enriched in dairy cows supplemented with NAM, and some serum polyamines (spermidine and putrescine), produced from the decarboxylation of arginine/ornithine in ruminants, changed [3]. Most AA concentrations were elevated in this study, suggesting that RPN increased available AA for potential tissue utilization.

Moreover, plasma metabolomics showed that the class of metabolites was mainly coordinated to AA and its metabolites, benzene, and substituted derivatives in the RPN group compared with the CON group. Interestingly, we found that small peptides, such as Ala-Asn-Arg-Val-Thr, Leu-Thr-Gln-Gln-Leu, Glu-Asn-Ile-Ile-Asp, His-Phe-Arg-Asp, Lys-Thr-Glu-Lys-Ala, Val-Tyr-Gln-His-Val, and Tyr-His-Arg-Arg, were significantly decreased in the plasma of the RPN group. The VIP values of these small peptides were higher than 2, suggesting that RPN supplementation plays an important role in small peptide metabolism. The dietary protein in the intestine is mainly absorbed in the form of free AAs and small peptides and delivered to various tissues through the blood for protein synthesis and energy metabolites [31]. Currently, based on their more effective and energy-saving features, peptide-bound AA has drawn increasing attention compared to free AA. Multiple types of small peptides were found to be absorbed through the intestinal peptide transporter PepT1 [32], and PepT1 is primarily expressed in the intestinal epithelial cells of the gastrointestinal tract of cows [33]. Many factors can modulate PepT1 expression and function, such as substrates, proteins, hormones, and diseases [34]. The circulating peptides may not be hydrolysed in plasma and are transported into cells then hydrolysed into free AA [35]. The plasma AA and peptide concentrations were governed by the balance between AA and peptide supply from the gut and utilization or catabolism by the tissues. Thus, we speculated that the reasons for the increased AA and decreased peptide concentrations by RPN supplementation would be i) the absorption of peptides in the intestine might be lower than that of AAs and (or) ii) the utilization of peptides might be more than that of AAs. Further study will focus on determining the reasons. We also found that nitrogen metabolism was enriched in this study, while ruminal NH_3_-N, BUN, and blood ammonia were not affected, indicating that RPN did not induce a poor balance of plasma AA and disarray of AA metabolism. In addition, as suggested by Toledo et al [36], compared to mixing with TMR, the top dressing helped animals consume most supplements immediately after feeding, leading to a clear peak of the corresponding AA in plasma. In general, NAD is essential in many enzymatic reactions as a cofactor and plays important roles in energy metabolism [37]; therefore, increased NAM resulted in increased overall energy metabolism in this study.

## CONCLUSION

Supplementing rumen-protected nicotinamide to lambs favoured the rumen fermentation pattern to propionic acid-type and benefitted their health. The results presented in this study provided the first evidence that rumen-protected nicotinamide supplementation altered the amino acid profile. More research is warranted to examine the mechanism of nicotinamide on amino acid and peptide circulation.

## Figures and Tables

**Table 1:** Nutrient contents of the pelleted total mixed rations (g/kg of DM, unless noted)

Nutrient analyses	Values
DM (g/kg of fresh)	867.4
CP	166.2
NDF	230.8
ADF	96.5
Ether extract	48.3
Ca	11.8
P	5.3
Ash	53.6
GE (MJ/kg)	13.46

DM, dry matter; CP, crude protein; NDF, neutral detergent fibre; ADF, acid detergent fibre; Ca, calcium; P, phosphorus; GE, gross energy.

**Table 2:** Effect of rumen-protected nicotinamide supplementation on the growth of growing lambs

Items	Treatments^1)^				SEM	p-value		
	
	T0	T0.5	T1	T2		Treat	Linear	Quadratic
Body weight (kg)								
Day 70	21.30	20.89	21.57	21.38	0.283	0.877	0.708	0.380
Day 100	28.88	29.74	28.90	28.57	0.277	0.570	0.515	0.666
Day 130	38.44	39.40	37.72	38.12	0.257	0.128	0.320	0.318
Day 160	42.81	44.50	42.79	44.00	0.403	0.369	0.553	0.845
Average daily gain (g)								
Day 70-100	239.9	242.2	241.5	226.8	5.80	0.785	0.428	0.408
Day 100-130	335.7	327.2	326.3	311.3	6.90	0.717	0.257	0.479
Day 130-160	162.0	146.5	194.8	195.9	11.64	0.387	0.161	0.380
Day 70-130	291.9	291.7	285.3	269.1	3.72	0.065	0.014	0.054
Day 70-160	236.1	259.6	243.2	245.9	3.84	0.161	0.583	0.315
Day 100-160	234.2	220.1	242.0	257.0	6.35	0.234	0.139	0.242
Feed efficiency								
Day 70-100	4.54	4.58	4.64	4.90	0.111	0.702	0.257	0.284
Day 100-130	4.69	4.58	4.97	4.86	0.089	0.454	0.281	0.505
Day 130-160	10.86	13.03	8.90	10.01	0.604	0.066	0.231	0.483

SEM, standard error of the mean; RPN, rumen-protected nicotinamide.

1) T_0_, T_0.5_, T_1_, and T_2_ represent growing lambs supplemented with 0, 0.5, 1, and 2 g/d RPN, respectively.

**Table 3:** Effect of rumen-protected nicotinamide supplementation on rumen fermentation in growing lambs

Items	Treatments^1)^				SEM	Contrast		
	
	T_0_	T_0.5_	T_1_	T_2_		Treat	Linear	Quadratic
Total VFA concentration (mM)	30.48^c^	48.49^b^	58.95^b^	74.59^a^	3.555	<0.001	<0.001	<0.001
VFAs proportions, % of total VFAs								
Acetic acid	60.47^a^	51.44^b^	49.45^b^	49.85^b^	1.25	0.003	0.001	0.034
Propionic acid	25.88^b^	31.96^ab^	38.08^a^	36.66^a^	1.52	0.017	0.004	0.180
Butyric acid	10.07	11.52	8.95	8.11	0.63	0.252	0.136	0.360
Isobutyric acid	1.21^a^	0.89^b^	0.64^b^	0.60^b^	0.06	0.001	0.000	0.193
Valeric acid	1.39^b^	1.94^a^	2.39^a^	2.02^a^	0.10	0.001	0.002	0.007
Isovaleric acid	0.89^a^	0.70^a^	0.40^b^	0.30^b^	0.06	<0.001	0.000	0.570
Isoacids	3.58	3.51	3.62	3.42	0.17	0.978	0.821	0.850
A:P ratio	2.35^a^	1.83^ab^	1.28^b^	1.55^ab^	0.133	0.035	0.013	0.119
NH_3_-N, (mg/100 mL)	1.81	1.71	1.58	1.47	0.072	0.394	0.103	0.720
MCP (mg/L)	504.8	514.8	497.9	508.6	2.831	0.178	0.837	0.950

SEM, standard error of the mean; VFAs, volatile fatty acids; NH_3_-N, ammonia nitrogen; MCP, microprotein.

1) T_0_, T_0.5_, T_1_, and T_2_ represent growing lambs supplemented with 0, 0.5, 1, and 2 g/d rumen-protected nicotinamide, respectively.

a–c Means within a row with different superscripts differed (p<0.05).

**Table 4:** Effect of rumen-protected nicotinamide supplementation on blood parameters in growing lambs

Items	Treatments^1)^				SEM	Contrast		
	
	T_0_	T_0.5_	T_1_	T_2_		Treat	Linear	Quadratic
NAM (μg/L)	54.35^d^	73.59^c^	90.96^b^	104.02^a^	3.308	<0.001	<0.001	<0.001
T-AOC (mM)	0.15^b^	0.23^a^	0.27^a^	0.14^b^	0.011	<0.001	0.806	<0.001
SOD (U/mL)	94.28^b^	105.87^ab^	118.28^a^	125.03^a^	3.865	0.023	0.002	0.733
CAT (U/mL)	1.48	1.89	2.29	2.07	0.127	0.148	0.056	0.205
MDA (μM)	3.33	4.27	3.30	3.72	0.222	0.384	0.914	0.569
Glucose (mM)	5.39^b^	5.92^ab^	6.30^a^	6.60^a^	0.159	0.038	0.005	0.696
TG (mM)	0.509	0.478	0.646	0.486	0.028	0.106	0.684	0.229
TC (mM)	1.85	1.86	1.98	1.79	0.044	0.492	0.859	0.246
NEFA (mM)	0.33	0.29	0.38	0.39	0.015	0.113	0.075	0.460
Total protein (g/L)	71.94^b^	76.14^ab^	82.30^a^	75.54^b^	1.193	0.021	0.093	0.016
BUN (mM)	5.37	6.35	6.40	5.86	0.197	0.230	0.382	0.058
Blood ammonia (μM)	448.3	480.6	484.7	473.9	7.856	0.385	0.245	0.188
D-lactate (mM)	4.00	4.45	4.45	3.47	0.187	0.186	0.334	0.058
DAO (U/L)	5.16	4.40	5.28	5.70	0.262	0.356	0.298	0.269

SEM, standard error of the mean; NAM, nicotinamide; T-AOC, total antioxidant capacity; SOD, superoxide dismutase; CAT, catalase; MDA, malondialdehyde; TG, triglycerides; TC, total cholesterol; NEFA, nonesterified fatty acid; BUN, blood urea nitrogen; DAO, diamine oxidase.

1) T_0_, T_0.5_, T_1_, and T_2_ represent growing lambs supplemented with 0, 0.5, 1, and 2 g/d rumen-protected nicotinamide, respectively.

a–d Means within a row with different superscripts differed (p<0.05).

**Table 5:** Effect of rumen-protected nicotinamide supplementation on the plasma concentrations of amino acids in growing lambs

Items (μmol/100 mL)	Treatments^1)^				SEM	Contrast		
	
	T_0_	T_0.5_	T_1_	T_2_		Treat	Linear	Quadratic
Asparagine	44.08^b^	44.37^b^	45.45^ab^	46.42^a^	0.323	0.035	0.005	0.568
Serine	7.52^c^	7.72^bc^	7.79^b^	8.66^a^	0.095	<0.001	<0.001	0.077
Glutamine	43.60^b^	43.71^b^	45.04^b^	46.97^a^	0.333	<0.001	<0.001	0.089
Proline	33.97^c^	34.41^c^	36.80^b^	39.17^a^	0.444	<0.001	<0.001	0.119
Glycine	11.85^b^	12.24^b^	12.80^a^	13.06^a^	0.118	<0.001	<0.001	0.732
Alanine	8.07	8.00	8.01	8.15	0.038	0.488	0.478	0.179
Cysteine	8.23	8.18	8.38	8.46	0.047	0.121	0.038	0.454
Tyrosine	8.17	8.13	7.90	8.09	0.053	0.263	0.294	0.270
Valine	19.78^b^	20.10^b^	21.98^a^	20.46^b^	0.202	<0.001	0.008	0.005
DL-Methionine	19.73	19.71	20.25	20.35	0.148	0.276	0.078	0.826
Isoleucine	4.95^b^	5.02^b^	5.25^a^	5.26^a^	0.038	0.002	<0.001	0.651
Leucine	14.03	14.24	14.53	14.42	0.092	0.264	0.089	0.376
Threonine	33.92^b^	34.12^b^	35.29^ab^	36.46^a^	0.333	0.019	0.003	0.425
Phenylanaline	7.25^b^	7.15^b^	7.49^a^	7.66^a^	0.050	<0.001	<0.001	0.082
Lysine	29.80^b^	30.40^b^	33.21^a^	33.54^a^	0.339	<0.001	<0.001	0.758
Histidine	37.84	36.92	37.13	37.27	0.193	0.402	0.391	0.176
Arginine	157.30^b^	157.08^b^	162.22^ab^	166.46^a^	1.323	0.027	0.005	0.364
Total amino acids	490.11^c^	491.50^c^	509.68^b^	520.86^a^	2.548	<0.001	<0.001	0.118

SEM, standard error of the mean.

1) T_0_, T_0.5_, T_1_, and T_2_ represent growing lambs supplemented with 0, 0.5, 1, and 2 g/d rumen-protected nicotinamide, respectively.

a–c Means within a row with different superscripts differed (p<0.05).
